# Fear of childbirth, nonurgent obstetric interventions, and newborn outcomes: A randomized controlled trial comparing mindfulness‐based childbirth and parenting with enhanced care as usual

**DOI:** 10.1111/birt.12571

**Published:** 2021-07-11

**Authors:** Irena K. Veringa‐Skiba, Esther I. de Bruin, Francisca J. A. van Steensel, Susan M. Bögels

**Affiliations:** ^1^ Research Institute of Child Development and Education (RICDE) Research Priority Area Yield University of Amsterdam Amsterdam the Netherlands; ^2^ UvA‐minds Academic Center of the University of Amsterdam Amsterdam the Netherlands

**Keywords:** cesarean birth, fear of childbirth, mindfulness

## Abstract

**Objective:**

To investigate whether mindfulness‐based childbirth and parenting (MBCP) or enhanced care as usual (ECAU) for expectant couples decreases fear of childbirth (FOC) and nonurgent obstetric interventions during labor and improves newborn outcomes.

**Design:**

Randomized controlled trial.

**Setting:**

Midwifery settings, the Netherlands, April 2014‐July 2017.

**Population:**

Pregnant women with high FOC (n = 141) and partners.

**Methods:**

Allocation to MBCP or ECAU. Hierarchical multilevel and intention‐to‐treat (ITT) and per‐protocol (PP) analyses.

**Main outcome measures:**

Primary: pre‐/postintervention FOC, labor anxiety disorder, labor pain (catastrophizing and acceptance), and preferences for nonurgent obstetric interventions. Secondary: rates of epidural analgesia (EA), self‐requested cesarean birth (sCB), unmedicated childbirth, and 1‐ and 5‐minute newborn's Apgar scores.

**Results:**

MBCP was significantly superior to ECAU in decreasing FOC, catastrophizing of labor pain, preference for nonurgent obstetric interventions, and increasing acceptance of labor pain. MBCP participants were 36% less likely to undergo EA (RR 0.64, 95% CI [0.43‐0.96]), 51% less likely to undergo sCB (RR 0.49, 95% CI [0.36‐0.67]), and twice as likely to have unmedicated childbirth relative to ECAU (RR 2.00, 95% CI [1.23‐3.20]). Newborn's 1‐minute Apgar scores were higher in MBCP (DM −0.39, 95% CI [−0.74 to −0.03]). After correction for multiple testing, results remained significant in ITT and PP analyses, except EA in ITT analyses and 1‐minute Apgar.

**Conclusions:**

MBCP for pregnant couples reduces mothers’ fear of childbirth, nonurgent obstetric interventions during childbirth and may improve childbirth outcomes. MBCP adapted for pregnant women with high FOC and their partners appears an acceptable and effective intervention for midwifery care.

## 1 INTRODUCTION

The World Health Organization (WHO) has asked for a reduction in the use of nonurgent obstetric interventions during childbirth, such as the use of unnecessary cesarean birth (CB).^1^ Nonclinical interventions supporting this call are required^1^ to improve the health of mothers and newborns^2^ and to reduce health care costs.^3^ Common, nonurgent obstetric interventions include epidural analgesia (EA) and CB.^1^, ^4^, ^5^, ^6^, ^7^ Proportions of childbirths incorporating EA are 77% in France,^8^ 73% in USA,^9^ and 44% in Sweden.^10^ CB is requested by 28% of pregnant women in China^4^ and Brazil^5^ and 10% in Norway.^7^ Half of all births in China and Brazil,^4^ and a third of all births in the United States^11^ result in CB, whereas in the Netherlands, only 15% of all births are by CB.^12^ Nevertheless, in the Netherlands, 60% of pregnant women starting labor in midwifery‐led care are referred to obstetricians for nonurgent obstetric interventions, resulting from a failure to progress or an inability to cope with labor pain.^12^, ^13^

Although EA and CB are valued obstetric achievements, they are not risk‐free. For example, EA is associated with assisted vaginal births^6^ and a lower Apgar score in newborns.^10^, ^14^ The risk of severe acute maternal morbidity is five times higher with CB than with vaginal births.^15^ Furthermore, having had a previous CB increases the risk for morbidity in ongoing pregnancy by three times.^16^ Children born by CB have an increased risk of allergo‐immunological problems, asthma, and obesity.^2^

Worldwide, self‐requested CB (sCB) and EA are strongly associated with a fear of childbirth (FOC),^1^, ^17^, ^18^ and a fear of pain.^4^, ^5^ FOC is a complex concept incorporating different aspects of fear and anxiety within and external to the pregnancy itself.^19^, ^20^ Untreated FOC is a risk factor for traumatic childbirth,^18^, ^21^ and pregnancy specific anxiety—including fear of birth—is associated with impaired neuro‐emotional development in newborns caused by high levels of maternal cortisol.^22^, ^23^ Reducing FOC may reduce sCB and EA; however, scarce research on the use of nonclinical interventions to reduce CB rates currently exists.^24^ Psychoeducation is a nonclinical intervention associated with less FOC,^25^ and a birth plan is associated with better childbirth outcomes.^26^ Mindfulness‐based interventions (MBIs) are nonclinical interventions aimed at reducing symptoms like anxiety^27^ and chronic pain.^28^ Therefore, MBI might also be beneficial to reduce FOC. Pooled results of uncontrolled studies and (underpowered) RCT’s evaluating anxiety, depression, and perceived stress have demonstrated a significant benefit for different MBIs when compared with a control group.^29^ We conducted an adequately powered controlled study to investigate whether mindfulness‐based childbirth and parenting (MBCP) for pregnant women with high FOC, and their partners, would decrease FOC, as well as the use of EA and sCB, and improve childbirth outcomes, when compared with an active comparison group.

## 2 METHODS

### 2.1 Study design

We conducted a block randomized controlled trial (RCT) with two conditions: MBCP and enhanced care as usual (ECAU). The study involved screening with the Wijma‐Delivery Expectation Questionnaire^30^ (W‐DEQ‐A) before allocation (T0) and at three assessment time‐points: (1) 1 to 2 weeks pre‐intervention (T1 = 16‐26 weeks’ pregnancy), (2) postintervention (T2 = 26‐36 weeks’ pregnancy), and (3) medical data from childbirth reports (T3 = 2‐4 weeks’ postpartum). Recruitment took place between April 2014 and July 2017, facilitated by caregivers.

### 2.2 Participants

We included low‐risk, nulli‐, and multi‐parous pregnant women aged ≥18 years without a priori restriction on having an unmedicated childbirth (spontaneous, without any obstetric intervention), experiencing a high FOC (W‐DEQ‐A ≥ 66 and self‐confirmed FOC). Participants were recruited from midwifery care settings in Amsterdam and The Hague, the Netherlands. Exclusion criteria were unwillingness to be randomized, current severe psychological problems, participation in another MBP, or hypno‐birthing training in the past year. The use of stable‐dose antidepressant medication, participation in an ongoing psychological intervention, or a prenatal educational course were not exclusion criteria. Details about the recruitment procedure and inclusion and exclusion criteria can be found in the trial's protocol.^31^

### 2.3 Randomization and masking

Both conditions were presented to the referrers and pregnant women. The first author checked for randomization eligibility and screened participants at T0. An independent assistant communicated the allocation and sent the link per e‐mail for the precondition measurements at T1. Allocation was done according to the order of entry, using blocks of codes created using Microsoft Excel (Microsoft Corporation, Redmond, WA, USA). The blocks of codes started with MBCP, alternating between four to six participants, depending on recruitment speed, followed by ECAU. Although conducting T1 assessments before randomization is typical in RCTs, the decision for a priori allocation was based on a steadily increasing gestational age, dependence on recruitment speed and efficiency, required minimum group size, and adherence to an equal length of time (maximum 2 weeks) between T1 assessment and the start of MBCP/ECAU. In addition, the participants’ preferences for MBCP or ECAU were collected. The allocation process was concealed from the referrers and from the independent outcome assessor. Once allocated, conditions could not be concealed from the participants or referrers any longer. Data collection was carried out online, using required responses via the Qualtrics software (Qualtrics, Provo, UT, USA).

### 2.4 Experimental intervention

The experimental condition comprised the secular, face‐to‐face, group‐based MBCP program for expectant couples published as “Mindful Birthing.”^32^ We adapted the program for pregnant women with FOC, focusing on management techniques for anxiety and fear, guided meditations, and enquiry. The nine weekly sessions lasted three hours each and were delivered by experienced midwives certified in MBCP. Sessions included mindfulness meditation practice and enquiry (eg, participants sharing about meditation experiences to improve meditation practice), and teachings about psychobiological processes in the perinatal period for women, newborns, and the family.

Mindful meditation aims to cultivate the deliberate, immediate, and nonjudgmental quality of attention to current experiences. This quality of attention allows individuals to observe experiences (such as physical sensations, thoughts, and emotions) through a gentle lens, resulting in increased tolerance and acceptance, and reduced reactivity to these experiences.^33^ Participants were asked to commit to meditation practices at home for 30 minutes each day.

### 2.5 Enhanced care as usual

ECAU^34^ consisted of two individual 90‐minute sessions for the expectant couple. Both sessions were spread over a ten‐week period (like MBCP) and were delivered by trained midwives. ECAU was designed to reduce FOC by gaining insight into the factors causing and maintaining fear and stress in the perinatal period, including psychoeducation about fear, and making a coping plan. The first session was based on the biopsychosocial model,^35^ and the second session consisted of writing the Childbirth Plan of the Royal Dutch Organization of Midwives (KNOV).^36^ The content of the original MBCP and ECAU is described in more detail in the study protocol.^31^

### 2.6 Primary outcomes

The primary outcomes were FOC, labor anxiety disorder, labor pain (catastrophizing and acceptance), and preferences for nonurgent obstetric interventions in childbirth. FOC was measured using the 33‐item W‐DEQ‐A covering general fear, negative appraisal, loneliness, lack of self‐efficacy, lack of positive anticipation, and concerns about the child.^30^ Higher scores indicate increased FOC: high (W‐DEQ‐A ≥ 66), severe (W‐DEQ‐A ≥ 85), and phobic (W‐DEQ‐A ≥ 100).^37^ Labor anxiety disorder was assessed by the 10‐item subscale of the newly developed DSM‐5 Perinatal Anxiety Disorder‐Labor (DSM‐5 PAD‐L).^38^ Catastrophizing and acceptance of labor pain were assessed using the 12‐item Catastrophizing Labor Pain (CLP)^39^ and the 20‐item Labor Pain Acceptance Questionnaire (LPAQ).^40^ Pregnant women's preferences for nonurgent obstetric interventions were assessed using the 7‐item Willingness to Accept Obstetric Interventions (WAOI)^41^; scores >28 indicate preference for nonurgent obstetric interventions such as EA and CS. The Perinatal Disaster Scenario Scale (PDSS) was excluded from our analysis due to low responses (n = 53; 37.6%).

### 2.7 Secondary outcomes

The secondary outcomes in the pregnant women (rates of EA, sCB, and unmedicated childbirth [birth without obstetric intervention]) and the newborns (1‐ and 5‐minute Apgar score) were derived from medical files.

### 2.8 Sample size calculation

A priori power calculations showed that under the assumption of a medium effect size of MBCP compared with ECAU, at least 128 participants were required to achieve a power of 80% to find a significant effect (test of between‐within interaction, 5% alpha and 0.5 correlation).

### 2.9 Quality control

MBCP sessions were recorded and supervised by SB. ECAU sessions were audiotaped, and the birth plan was documented. Treatment acceptability in MBCP was assessed by registering session attendance and minutes spent practicing meditation exercises. In both groups, attendance at additional prenatal educational courses was registered.

### 2.10 Statistical analyses

**FIGURE 1:**
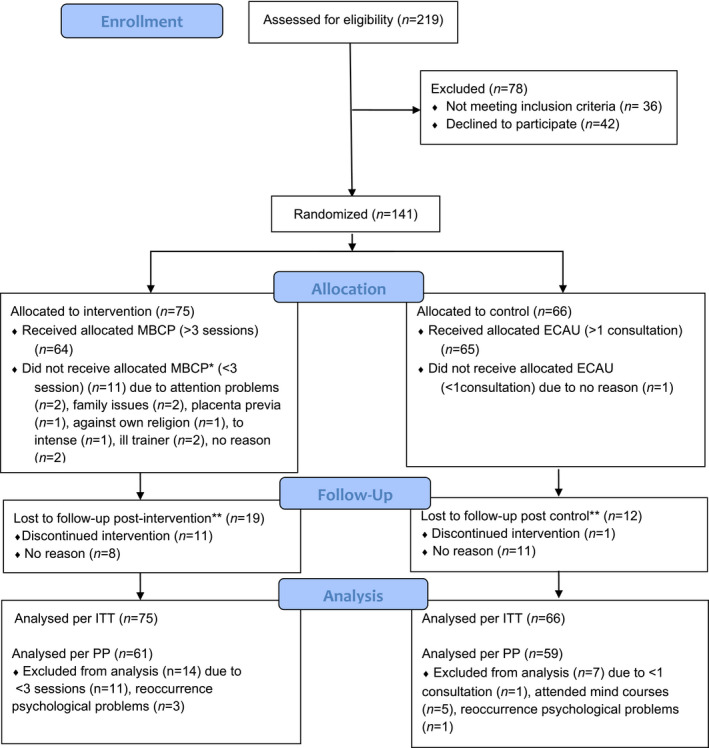
CONSORT 2010 Transparent Reporting of Trials: Flow Diagram ECAU, Enhanced Care As Usual; ITT, Intention to treat; MBCP, Mindfulness‐Based Childbirth and Parenting; PP, Per protocol. *Note:* *No statistically significant difference in the W‐DEQ‐A scores at T1 between participants who did receive a minimum intervention dose and those who did not (*t*(139) = −0.83; *P* = .83). **No statistically significant difference in lost‐to‐follow‐up between groups (*X*2 = 1.05, *P* = .31)

The primary analysis was performed using intention‐to‐treat (ITT). Little's Missing Completely at Random (MCAR) were used to identify missing data. We used hierarchical linear model (HLM) analyses for continuous outcomes; pre‐ and postassessments (level 1) were nested in individuals (level 2). We used fixed parameters and entered T2 (time 2; postintervention compared with pre‐intervention), condition (MBCP compared with ECAU, a main effect), and the interaction (T2*condition, a difference between effects) as predictors. T2 as a significant predictor indicated a main effect of time (ie, scores changed between pre‐ and postintervention). Condition as a significant predictor indicated a main effect of condition (a significant difference in scores between MBCP and ECAU). A significant interaction term indicated a difference between effects of MBCP and ECAU. All outcome measures were standardized. As such, parameter estimates could be interpreted as an effect size (Cohen's *d*: 0.2 = small, 0.5 = moderate, and 0.8 = large).^42^ Chi‐square analyses with Fisher's exact test were used for binary outcomes. Relative risk (RR) and relative risk reduction (RRR) with a 95% confidence interval (CI) were examined. Number needed to treat (NNT) with a 95% CI was calculated using MedCalc statistical software (MedCalc Software Ltd, Ostend, Belgium). Independent *t‐*tests were used for continuous outcomes that were assessed postintervention only (newborn outcomes). Analyses were also conducted per‐protocol using the same statistical rules (PP; Figure 1). All analyses were performed two‐sided, *α‐* level of 0.05, using SPSS (version 24; IBM Corp., Armonk, NY, USA). Since multiple tests were conducted, a Holm‐Bonferroni correction^43^ was applied to the obtained *P‐*values of primary and secondary outcomes to prevent type I errors. Standardized values > (−)3.29 were considered as outliers. With respect to HML analyses, no outliers were identified. Outliers in childbirth outcomes were not removed; two outliers for low Apgar at 1 minute, four outliers for low Apgar at 5 minutes, and two outliers for gestational age were found. Skewness and kurtosis values were within the boundary of −1.96 and 1.96, except for the Apgar 1‐ and 5‐minute scores and gestational age. Therefore, nonparametric tests were also run on these variables, which yielded similar results.

## 3 RESULTS

### 3.1 Recruitment and attrition

The rates of recruitment, reasons for refusal, exclusion, withdrawal, and attritions are summarized in the trial's flowchart (Figure 1). We randomly assigned 141 pregnant women to MCBP (n = 75) or ECAU (n = 66). To create equal numbers of participants who received a minimum intervention dose (as stated in the protocol^31^), we needed to recruit additional participants for MBCP to protect power. W‐DEQ‐A scores at T0 were similar between conditions (*P* = .45), as well as baseline characteristics (Table 1), and no differences were found between the participants who did (n = 113) and did not (n = 28) complete T2 measurements (*P* > .10). Missing data at T2 was random (MCAR test *χ* ^2^ = 12.70, *df* = 13, *P* = .47). No reporting bias was found because no difference in mean scores at T1 was revealed for the participants who were allocated to their preferred (n = 63) or nonpreferred (n = 50) condition (*P* > .50; n = 28 reported no preference). Notably, three‐quarters of the sample experienced previous psychological problems and one‐quarter was treated with medication for longer than a year. In both groups, about 85% of partners participated (*P* = .94).

**TABLE 1:** Baseline characteristics of participants for the intention‐to‐treat population at pre‐assessment (T1)

	MBCP (n = 75)	ECAU (n = 66)	*P*
Demographic characteristics			
Age, mean (SD)	33.11 (3.92)	32.72 (3.86)	.55
Ethnic origin, n (%)			
White	57 (76.0)	41 (62.1)	.19
Other	17 (22.7)	25 (37.9)	
Missing	1 (1.3)	–	
Education level, n (%)			
High	61 (81.3)	50 (75.8)	.19
Middle to low	11 (14.7)	16 (24.2)	
Missing	3 (4.0)	–	
Employment, n (%)			
Yes	64 (85.3)	51 (77.3)	.16
No	10 (13.3)	15(22.7)	
Missing	1 (1.4)	–	
Married/leaving together (yes), n (%)	68 (90.7)	65 (98.5)	.05
Partner participated in intervention (yes), n (%)	64 (85.3)	56 (84.8)	.94
Obstetric characteristics			
Parity (n, %)			
Nulliparous	51 (68.0)	38 (57.6)	.20
Multiparous	24 (32.0)	28 (42.4)	
Echelon of care (n, %)			
Midwife‐led care (yes)	66 (88.0)	59 (89.4)	.80
Obstetrician‐led care (yes)	9 (12.0)	7 (10.6)	
Anamnesis (n, %)			
Caesarean birth in history (yes)	4 (5.3)	7 (10.6)	.24
Intrauterine fetal death in history (yes)	4 (5.3)	1 (1.5)	.22
Current labor^a^			
Gestational age in weeks mean (SD)	39.43(1.73)	39.52 (1.44)	.75
Induction (n, %) (yes)	11(15.9)	9 (16.1)	.98
Dilatation period in hours mean (SD)	8.09 (5.45)	7.81 (5.05)	.80
Mental health characteristics			
W‐DEQ‐A, mean (SD)	94.72 (19.55)	92.33 (17.35)	.45
Psychological/psychiatric care in history (yes), n (%)	56 (74.7)	50 (75.8)	.88
Psychological/psychiatric care present (yes), n (%)	13 (17.3)	15 (22.7)	.44
Missing	2 (2.7)	1 (1.5)	
Medication for psychological problems >1 y (yes), n (%)			
Past	23 (30.7)	14 (21.2)	.20
Present	3 (4.0)	2 (3.0)	
Psychiatric hospitalization in history (yes), n (%)	4 (5.3)	2 (3.0)	.50

Abbreviations: ECAU, Enhanced Care As Usual; MBCP, Mindfulness‐Based Childbirth and Parenting; W‐DEQ‐A, Wijma Deliver Expectation Questionnaire.

^a^ Sample without primary caesarean birth (n = 14).

### 3.2 Quality control

Adherence to MBCP (following ITT) was assessed by the number of sessions attended (mean 6.8 ± 2.85; 87% attended four to nine sessions, 21% attended all nine sessions) and time spent on formal meditation practices per week (mean 85.05 ± 58.96 minutes). No significant difference was observed in W‐DEQ‐A scores at T1 between participants who received a minimum intervention dose (98.81 ± 22.10) and those who did not (89.92 ± 23.20; *t*(139) = −0.83, *P* = .83). Adherence to ECAU was also assessed by the number of consultations attended (98% followed at least one of the two sessions). In the ECAU group, significantly more (*P* < .001) pregnant women followed a prenatal educational course (41%; n = 27) than in MBCP (9%, n = 7). In addition, mindfulness awareness was assessed using the Five Facet Mindfulness Questionnaire (FFMQ)^44^ in both conditions. HLM analyses showed that mindfulness awareness only increased in MBCP (T2 = (−)0.17; condition = 0.03; T2*condition = 0.77; *SE* = 0.17, *P* < .001).

### 3.3 Primary outcomes

Tables 2 and 3 summarize the results of the HLM of the primary outcome as a function of time (T2 versus T1), intervention (condition MBCP versus ECAU), and interaction between time and intervention (T2*condition). Fear of childbirth mean scores (assessed by W‐DEQ‐A) decreased after MBCP and ECAU (significant effect for T2), but the decrease was significantly larger for MBCP (significant interaction T2*condition). To explore the clinical effect of this finding, total W‐DEQ‐A scores were dichotomized into normal and high (≥66).^37^ The risk of a high W‐DEQ‐A score at T2 was 36% lower after MBCP compared with ECAU (RR 0.64, 95% CI [0.45‐0.91], *P* = .01; RRR 36%, 95% CI [9%‐55%]). MBCP needs to be offered to five pregnant women to decrease FOC to a normal level in one pregnant woman (NNT 4.5, 95% CI [2.5‐20.3]). Labor anxiety disorder (assessed by DSM‐5 PAD‐L) did not change between pre‐ and postassessment, nor was there a significant difference between conditions. Catastrophizing labor pain (assessed by CLP) decreased significantly after MBCP and ECAU; however, participants receiving MBCP showed a significantly larger decrease than those receiving ECAU. Labor pain acceptance (assessed by LPAQ) increased significantly after MBCP and ECAU but increased significantly more for MBCP than for ECAU. Preferring nonurgent obstetric interventions (assessed by WAOI) did not change in ECAU, but decreased significantly in MBCP. To explore the clinical effect of this, total WAOI scores were dichotomized (cutoff ≥28).^41^ MBCP participants were 40% less likely to prefer nonurgent obstetric interventions than ECAU participants (RR 0.60, 95% CI [0.41‐0.88], *P* = .04; RRR 40%, 95% CI [12%‐59%]). At T1, 35% (n = 26/74) of MBCP and 28% (n = 18/65) of ECAU preferred sCB as the mode of delivery (*P* = .30). At T2, 14% (n = 8/57) of the MBCP and 53% (n = 20/53) of the ECAU participants preferred sCB as the mode of delivery (*P* < .001). MBCP needs to be offered to seven pregnant women to change this preference in one pregnant woman (NNT 7, 95% CI [3.7‐77.1]). Similar results as in ITT‐analyses were found in PP‐analyses (see Table S1). Significant findings in both analyses remained after *P*‐value adjustment (see Table 3 and S1).

**TABLE 2:** Hierarchical multi‐level analyses of the primary outcomes for the intent‐to‐treat population with time, condition (MBCP versus ECAU) and the interaction (time*condition) as predictors

	Parameter estimate	Standard error	*t*	*P*	*P’*	95% CI lower upper	
W‐DEQ‐A							
T2^a^	−0.68	0.11	−6.43	<.001		−0.80	−0.40
Condition^b^	−0.01	0.14	−0.04	.97		−0.29	0.28
T2*Condition	−0.41	0.15	−2.74	.01	.020	−0.70	−0.10
DSM−5 PAD−L							
T2^a^	−0.20	0.15	−1.33	.19		−0.49	0.10
Condition^b^	−0.15	0.20	−0.76	.45		−0.53	0.24
T2*Condition	−0.21	0.21	−1.04	.30	.300	−0.62	0.19
CLP							
T2^a^	−0.49	0.11	−4.61	<.001		−0.69	−0.28
Condition^b^	−0.06	0.15	−0.43	.67		−0.36	0.23
T2*Condition	−0.52	0.15	−3.57	.001	.005	−0.81	−0.23
LPAQ							
T2^a^	0.33	0.12	2.75	.01		0.09	0.56
Condition^b^	−0.03	0.16	−0.16	.87		−0.35	0.30
T2*Condition	0.56	0.16	3.42	.001	.005	0.24	0.89
WAOI							
T2^a^	−0.02	0.11	−0.16	.87		−0.23	0.19
Condition^b^	0.09	0.18	0.51	.61		−0.27	0.45
T2*Condition	−0.48	0.15	−3.29	.001	.005	−0.76	‐0.19

Note

Outcome variables are standardized and as such parameter estimates can be interpreted as an effect size (Cohen's *d*).

Abbreviations: CLP, Catastrophizing Labor Pain; DSM‐5 PAD‐L, DSM‐5 Perinatal Anxiety Disorder‐Labor; LPAQ, Labour Pain Acceptance Questionnaire; WAOI, Willingness to Accept Obstetrical Interventions; W‐DEQ‐A, Wijma‐Delivery Expectation Questionnaire.

^a^ Postintervention as compared to pre‐intervention.

^b^ MBCP as compared to ECAU. Cohen (1992) reports the following intervals for *d*: 0.1‐0.2: small effect; 0.2‐0.5: medium effect; >0.8: large effect. *P*’ = *P*‐value after Holm‐Bonferroni correction.

**TABLE 3:** Pre‐ (T1) and postassessment (T2) scores on primary outcome measures and MBCP and ECAU effectiveness for the intention‐to‐treat population

	MBCP					ECAU				
	T1 M (SD)	n	T2 M (SD)	n	*d*	T1 M (SD)	n	T2 M (SD)	n	*d*
W‐DEQ‐A	97.99 (23.32)	75	69.26 (24.75)	57	1.20	98.12 (21.08)	66	79.61 (24.21)	56	0.82
DSM‐5 PAD‐L	26.76 (7.89)	63	23.53 (7.11)	47	0.43^a^	27.80 (7.84)	55	26.27 (5.58)	47	0.22
CLP	46.62 (12.13)	74	33.12 (10.50)	57	1.18	47.48 (10.93)	65	40.36 (13.35)	53	0.59
LPAQ	60.22 (10.77)	74	69.60 (8.25)	57	‐0.96	60.55 (8.52)	65	64.19 (9.44)	53	‐0.41
WAOI	23.01 (7.44)	70	19.25 (5.43)	55	0.57	21.82 (6.02)	60	22.12 (6.56)	51	‐0.05

Note

Due to a technical error in data transmission, a part of the DSM‐5 PAD‐L data was lost. Cohen (1992) reports the following intervals for *d*: 0.1‐0.2: small effect; 0.2‐0.5: medium effect; >0.8: large effect.

Abbreviations: CLP, Catastrophizing Labor Pain; DSM‐5 PAD‐L, DSM‐5 Perinatal Anxiety Disorder‐Labor; ECAU, Enhanced Care As Usual; LPAQ, Labour Pain Acceptance Questionnaire; MBCP, Mindfulness‐Based Childbirth and Parenting; WAOI, Willingness to Accept Obstetrical Interventions; W‐DEQ‐A, Wijma‐Delivery Expectation Questionnaire.

^a^ Cohens’ *d* is based on: (M T1‐M T2)/pooled SD.

### 3.4 Secondary outcomes

MBCP participants were 36% less likely to undergo EA (RR 0.64, 95% CI [0.43‐0.96], *P* = .03), and 51% less likely to undergo sCB (RR 0.49, 95% CI [0.36‐0.67], *P* = .01). The MBCP participants were twice as likely to undergo unmedicated childbirth relative to ECAU (RR 2.00, 95% CI [1.23‐3.20], *P* = .002). MBCP needs to be offered to five pregnant women to prevent one EA in labor (NNT 5.0, 95% CI [2.7‐39.5]), to nine women to prevent one woman from undergoing sCB (NNT 9.0, 95% CI [5.2‐36.8]), and to four women to result in one unmedicated childbirth (NNT 4.4, 95% CI [2.7‐12.9]). The 1‐minute Apgar score in newborns was higher in MBCP than ECAU (DM −0.39, 95% CI [−0.74 to −0.03], *P* = .03), but no difference was seen in the 5‐minute Apgar score (*P* = .28). Note, that after *P*‐values adjustment, MBCP and ECAU still differed significantly on the outcome variables “underwent sCB” and “unmedicated childbirth” (see Table 4), but no longer on “EA in labor” and “1‐minute APGAR score.”

**TABLE 4:** Differences in the secondary outcomes between the MBCP and ECAU for the intention‐to‐treat population

Childbirth outcome										
Pregnant women	MBCP (n = 75)	n^b^	ECAU (n = 66)	n	*χ* ^b^	*P*	*P’*	RR (95%CI)	RRR % (95%CI)	NNT (95% CI)
Used EA in labor (yes)^a^	27	69	33	56	4.854	.028	.084	0.64 (0.43‐0.96)	36 (4‐57)	5.0 (2.7‐39.5)
Underwent sCB (yes)	1	74	8	65	6.860	.009	.036	0.49 (0.36‐0.67)	51 (33‐64)	4.4 (2.6‐12.9)
Had an unmedicated birth (yes)	35	74	14	65	10.05	.002	.010	2.00 (1.23‐3.20)	**–**	3.9 (2.4‐9.6)

Newborns	M (SD)	n^b^	M (SD)	n^b^	*t*	*P*	*P’*	Mean difference	95%CI
1‐min APGAR score	9.03 (0.84)	71	8.64 (1.21)	64	−2.17	.030	.084	−0.38	−0.74‐(−)0.03
5‐min APGAR score	9.76 (0.64)	71	9.61 (0.92)	64	−1.12	.280	.280	−0.15	−0.42‐0.12

Abbreviations: EA, Epidural Analgesia; ECAU, Enhanced Care As Usual; MBCP, Mindfulness‐Based Childbirth and Parenting; sCB, self‐requested Caesarean Birth.

^a^ Sample without primary CB (n = 14)

^b^ Sample size depending on availability of medical files data. *P*’ = *P*‐value after Holm‐Bonferroni correction.

Similar results as in ITT‐analyses were found in PP‐analyses (see Table S2). Note that in PP analyses, all birth outcomes including “used EA in labor” remained significant after *P*‐value adjustment; however, “1‐minute Apgar score” was no longer significant.

## 4 DISCUSSION

### 4.1 Main findings

Our findings suggest that MBCP is more effective than ECAU in reducing FOC, catastrophizing labor pain, preferences for nonurgent obstetric interventions, and rates of self‐requested CB, and in increasing acceptance of labor pain, and unmedicated childbirth. This was found both in ITT and PP analyses and after *P*‐values adjustment. In addition, the PP analyses showed that MBCP participants used epidural analgesia less often than ECAU participants. Moreover, newborn's 1‐minute Apgar score was higher after MBCP than ECAU, but only in ITT analyses and without adjustment for multiple testing.

### 4.2 Strengths and limitations

The strengths of this study include the adequate statistical power, a real‐life active control group, the use of self‐reported (subjective) and childbirth measures (objective) derived directly from the medical files, the use of a study protocol, corroborative ITT and PP analyses, adjustment of *P*‐values for multiple testing to decrease type 1 errors and blinding of the outcome assessor to group allocations. Both conditions were presented as equal and delivered by trained midwives. Mean scores at both pre‐assessments did not differ between MBCP and ECAU, indicating successful randomization.

This study had the following limitations. First, we did not include self‐reported questionnaires to monitor possible adverse events. However, no problems were reported after the sessions, and therefore, it seems unlikely that there were clinically relevant adverse reactions. Second, due to the low number of occurrences of certain events (such as sCB), generalization is more difficult. Replication of the study, preferably with a larger sample size and in other cultures where sCB is more common, is required. Third, we cannot rule out that greater effect of MBCP (9 group couple sessions) compared with ECAU (2 individual couple consultations), both delivered within ten weeks, was due to a dose difference. However, it should be noted that significantly more participants in ECAU (41%) than in MBCP (9%) followed additional prenatal educational courses, which may have compensated for the dose difference. Fourth, pre‐intervention assessments were conducted after allocation, which could have caused a measurement bias due to knowing to which condition participants were allocated. However, we did not find evidence of baseline differences between conditions, and no differences between participants who were and were not allocated to their preferred condition were found. Finally, a substantial proportion of the postassessment data (24%) was missing. This could have impacted the results. However, participants with and without missing postassessment data did not differ on pre‐assessment measurements and overall missing data were at random. In addition, birth outcome data were retrieved from medical files also for those participants that did not complete the postassessment. Furthermore, the percentage of participants missing postassessment data seems to be common in studies on pregnant women with high FOC.^45^

### 4.3 Interpretation

Although mean pre‐assessment fear of childbirth scores (W‐DEQ‐A = 98) indicated almost phobic levels,^37^ both groups showed a substantial decrease. However, the MBCP participants were 40% less likely to report high FOC scores on the dichotomized post‐assessment measurement (W‐DEQ‐A ≥ 66) than the ECAU participants, demonstrating an even greater effect for MBCP. Similar findings in favor of MBCP were found for catastrophizing labor pain, acceptance of labor pain, and preferences for nonurgent obstetric interventions during childbirth. However, no difference between conditions was found on the newly developed scale assessing labor anxiety disorder, which could be explained by less power since 36% of this data was missing due to technical errors. This scale is not yet validated. Our finding of reduced FOC in MBCP corroborates evidence from several, mostly small, uncontrolled, and controlled (but largely underpowered) studies on the effects of different mindfulness‐based interventions on improvements in mental health conducted across different populations of pregnant women, care systems, and countries.^29^ Before the current study, there was no adequately powered RCT evaluating a mindfulness‐based intervention or MBCP on FOC (W‐DEQ‐A ≥ 66) and/or on childbirth outcomes. One well‐powered RCT showed that MBCP is more effective in decreasing perceived stress (*P* = .038, *d* = 0.30) and being at risk for perinatal depression (*P* = .004, *d* = 0.42) as compared with a Lamaze childbirth course.^46^ Furthermore, MBCP effects seem to be comparable to the effects of educational interventions reducing high FOC (W‐DEQ‐A ≥ 66; respectively, MD −0.41 and SMD −0.46).^47^ Lastly, only one RCT evaluated the effect of a nonclinical intervention on reducing CB. It was found that a childbirth training workshop (as compared to routine maternity care) reduced the number of CB (RR 0.59, 95% CI [0.37‐0.94]). However, this RCT was of low quality as evaluated by Cochrane,^24^ and therefore, caution about the interpretation of this result is needed.

In this study, the effectiveness of MBCP in reducing nonurgent obstetric interventions in labor (EA by almost 40%; sCB 50%) is promising. After *P*‐value adjustment for multiple testing, the reduced use of EA after MBCP compared with ECAU was no longer significant in ITT analysis; as such, this finding is somewhat uncertain. The low use of sCB in MBCP (1.4%) is particularly interesting given the relatively high percentage (35%) of participants in this group who *a prior* stated a preference for nonmedically indicated CB. The higher 1‐minute Apgar scores in newborns of mothers participating in MBCP could result from less intrapartum EA use^10^, ^14^ in MBCP (39%) compared with ECAU (59%) participants. However, results are somewhat uncertain as after *P*‐value adjustment this effect was no longer significant. In addition, Apgar scores in newborns 5 minutes after birth did not differ between the two groups. More research with larger samples is needed to draw more definite conclusions about the outcomes for newborns.

Considering the relatively low NNT, MBCP may be a promising intervention to ameliorate severe FOC and reduce nonurgent obstetric interventions such as sCB (and EA), and substantially increase the frequency of unmedicated childbirths. The relatively low rate of sCB in this study can be explained by the structure of Dutch midwifery health care system, which is based on the idea that pregnancy and childbirth are natural processes that occur under the care of midwives. This care system is designed to minimize medically unnecessary interventions of any kind. However, according to Dutch national data, 71% of pregnant women in 2018 gave birth with an obstetrician, and the rate of CB was 15%.^12^ The global rate of CB has doubled in the past 15 years to 21% and is increasing annually by 4%.^48^ The rate of CB exceeds 40% in at least 15 countries.^11^ The Dutch national cohort study concluded that: “compared to vaginal birth, maternal mortality after cesarean section was three times higher following exclusion of deaths that had no association with surgery.”^49^ Although CB is a relatively safe obstetric intervention, keeping the CS rate as low as possible should be in the interests of all pregnant women. The WHO has emphasized the need for nonclinical interventions to reduce unnecessary CB and to support unmedicated childbirth, for example, by tailoring information and support about FOC, pain relief, and the advantages and disadvantages of medical interventions in childbirth.^1^ Our research shows that MBCP could potentially contribute to achieving these goals.

### 4.4 Conclusions

Our findings suggest that offering mindfulness training to pregnant women suffering from high FOC and their partners is effective in decreasing FOC and nonurgent obstetric interventions such as sCB, and substantially increasing unmedicated childbirths. The nine‐week MBCP program adapted for pregnant women with high FOC and their partners appears an acceptable and effective intervention for midwifery care. The increase of FOC and use of nonurgent obstetric interventions during childbirth are worldwide concerns. Whether our findings have wider application deserves further study and attention from health care policy makers.

## CONFLICT OF INTEREST

The authors have no disclosures to declare. Completed disclosure of interest forms available to view online as supportive information.

## ETHICAL APPROVAL

The study protocol of the randomized controlled trial (I’ve Changed My Mind) was submitted to the Netherlands Trial Register (NTR; 4302) on December 3, 2013, before the study began and was accepted on January 03, 2014. The Ethics Review Board of the Faculty of Social and Behavioral Sciences at the University of Amsterdam approved the trial on 23 June 2013 (certificate number 2013‐CDE‐3064). The study was exempted from the approval by the Medical Ethical Committee of the Academic Medical Centre (certificate number NL44033.018.13).

## DATA AVAILABILITY STATEMENT

Data available on request from the authors.

## Supporting information

Table S1

Table S2
