# Supplementary material for: Fear of childbirth, nonurgent obstetric interventions, and newborn outcomes: A randomized controlled trial comparing mindfulness‐based childbirth and parenting with enhanced care as usual
Source: Birth. 2021 Jul 11;49(1):40–51. doi: 10.1111/birt.12571 (PMC9292241; doi:10.1111/birt.12571)
Supplement: Supplementary file 1 — Table S1 [file BIRT-49-40-s001.docx]

**Table S1.** Hierarchical multi-level analyses of the primary outcomes with time and condition as predictors between the MBCP and ECAU participants for the per-protocol population.

|  | **Parameter**  **estimate** | **Standard**  **error** | ***t*** | ***P*** | ***P’*** | **95% CI**  **lower upper** | |
| --- | --- | --- | --- | --- | --- | --- | --- |
| **W-DEQ-A** |  |  |  |  |  |  |  |
| T2^a^ | -0.62 | 0.11 | -5.58 | <0.001 |  | -0.83 | -0.40 |
| Condition^b^ | -0.01 | 0.15 | 0.05 | 0.96 |  | -0.30 | 0.31 |
| T2*Condition | -0.57 | 0.15 | -3.70 | <0.001 | <0.001 | -0.87 | -0.26 |
| **DSM-5 PAD-L** |  |  |  |  |  |  |  |
| T2^a^ | -0.27 | 0.15 | -1.85 | 0.07 |  | -0.56 | 0.02 |
| Condition^b^ | -0.28 | 0.21 | -1.33 | 0.19 |  | -0.70 | 0.14 |
| T2*Condition | -0·14 | 0.21 | -1·69 | 0.49 | 0.49 | -0.55 | 0.27 |
| **CLP** |  |  |  |  |  |  |  |
| T2^a^ | -0.43 | 0.11 | -3.95 | <0.001 |  | -0.65 | -0.21 |
| Condition^b^ | -0.09 | 0.16 | -0.55 | 0.58 |  | -0.40 | 0.23 |
| T2*Condition | -0.62 | 0.15 | -4.11 | <0.001 | <0.001 | -0.91 | -0.32 |
| **LPAQ** |  |  |  |  |  |  |  |
| T2^a^ | 0.36 | 0.12 | 2.91 | 0.004 |  | 0.11 | 0.61 |
| Condition^b^ | 0.04 | 0.18 | 0.25 | 0.81 |  | -0.31 | 0.39 |
| T2*Condition | 0.58 | 0.17 | 3.37 | 0.001 | 0.003 | 0.24 | 0.91 |
| **WAOI** |  |  |  |  |  |  |  |
| T2^a^ | 0.02 | 0.11 | 0.15 | 0.89 |  | -0.20 | 0.24 |
| Condition^b^ | 0.02 | 0.20 | 0.10 | 0.92 |  | -0.37 | 0.41 |
| T2*Condition | -0.53 | 0.15 | -3.47 | 0.001 | 0.003 | -0.84 | -0.23 |

CLP=Catastrophizing Labour Pain; DSM-5 PAD-L=DSM-5 Perinatal Anxiety Disorder-Labour; LPAQ=Labour Pain Acceptance Questionnaire; WAOI=Willingness to Accept Obstetrical Interventions; W-DEQ-A= Wijma-Delivery Expectation Questionnaire.

*Note:* Outcome variables are standardized and as such parameter estimates can be interpreted as an effect size (Cohen’s *d*). ^a^ post intervention as compared to pre-intervention, ^b^ MBCP as compared to ECU. Cohen (1992) reports the following intervals for *d*: 0.1-0.2: small effect; 0.2-0.5: medium effect; > 0.8: large effect.
