# Supplementary material for: Fear of childbirth, nonurgent obstetric interventions, and newborn outcomes: A randomized controlled trial comparing mindfulness‐based childbirth and parenting with enhanced care as usual
Source: Birth. 2021 Jul 11;49(1):40–51. doi: 10.1111/birt.12571 (PMC9292241; doi:10.1111/birt.12571)
Supplement: Supplementary file 2 — Table S2 [file BIRT-49-40-s002.docx]

**Table S2.** Differences in the secondary outcomes between the MBCP and ECAU for the per-protocol population.

|  | **MBCP (*n*= 61)** |  | **ECAU (*n* = 59)** |  |  |  | |  | | | |  |  |  |
| --- | --- | --- | --- | --- | --- | --- | --- | --- | --- | --- | --- | --- | --- | --- |
| **Childbirth outcome** |  |  |  |  |  |  |  | | | |  | |  |  |
| **Pregnant women** |  | ***n* ^2^** |  | ***n*** | ***χ^2^*** | ***P*** | ***P’*** | | **RR (95%CI)** | | | | **RRR % (95%CI)** | **NNT (95% CI)** |
| Used EA in labour (yes)^1^ | 18 | 58 | 32 | 50 | 11.737 | 0.001 | 0.004 | | | 0.48 (0.31 – 0.75) | | | 52 (25 – 69) | 3.0 (1.9 – 6.6) |
| Underwent sCB (yes) | 0 | 61 | 8 | 59 | 8.862 | 0.003 | 0.009 | | | 0.45 (0.37 – 0.56) | | | 55 (44 – 63) | 7.5 (4.5 – 22.8) |
| Had an unmedicated birth (yes) | 33 | 61 | 10 | 59 | 18.000 | <0.001 | <0.001 | | | 2.74 (1.55 – 4.83) | | | **-** | 2.7 (1.9 – 4.7) |
| **Newborns** | **M (SD)** | ***n* ^2^** | **M (SD)** | ***n* ^2^** | ***t*** | ***P*** | ***P’*** | | | **Mean difference** | | | **95%CI** |  |
| 1-minute APGAR score | 9.02 (0.83) | 58 | 8.62 (1.27) | 58 | -2.00 | 0.048 | 0.096 | | | -0.40 | | | -0.79 – 0.00 |  |
| 5-minute APGAR score | 9.78 (0.59) | 71 | 9.59 (0.96) | 58 | -1.25 | 0.20 | 0.20 | | | -0.19 | | | -0.48 – 0.10 |  |

EA = Epidural Analgesia; ECAU = Enhanced Care As Usual; MBCP = Mindfulness-Based Childbirth and Parenting; sCB = self-requested Caesarean Birth. *Note:* ^1^ Sample without primary CS (*n* = 14); **^2^** Sample size depending on availability of medical files data. *P*’= *P*-value after Holm-Bonferroni correction.
